# Exploring the role of NLRP3 inflammasome in diabetic nephropathy and the advancements in herbal therapeutics

**DOI:** 10.3389/fendo.2024.1397301

**Published:** 2024-07-22

**Authors:** Jiangyuan Jin, Mianzhi Zhang

**Affiliations:** ^1^ School of Graduate Studies, Tianjin University of Traditional Chinese Medicine, Tianjin, China; ^2^ Department of Nephrology, Tianjin Academy of Traditional Chinese Medicine Affiliated Hospital, Tianjin, China

**Keywords:** NLRP3 inflammasome, diabetic nephropathy, pathogenesis, traditional Chinese medicine, therapeutic intervention

## Abstract

Diabetic nephropathy (DN), a prevalent complication of diabetes mellitus (DM), is clinically marked by progressive proteinuria and a decline in glomerular filtration rate. The etiology and pathogenesis of DN encompass a spectrum of factors, including hemodynamic alterations, inflammation, and oxidative stress, yet remain incompletely understood. The NOD-like receptor pyrin domain-containing 3 (NLRP3) inflammasome, a critical component of the body’s innate immunity, plays a pivotal role in the pathophysiology of DN by promoting the release of inflammatory cytokines, thus contributing to the progression of this chronic inflammatory condition. Recent studies highlight the involvement of the NLRP3 inflammasome in the renal pathology associated with DN. This article delves into the activation pathways of the NLRP3 inflammasome and its pathogenic implications in DN. Additionally, it reviews the therapeutic potential of traditional Chinese medicine (TCM) in modulating the NLRP3 inflammasome, aiming to provide comprehensive insights into the pathogenesis of DN and the current advancements in TCM interventions targeting NLRP3 inflammatory vesicles. Such insights are expected to lay the groundwork for further exploration into TCM-based treatments for DN.

## 1 Introduction

Diabetic nephropathy (DN) represents a severe complication of diabetes mellitus (DM), emerging as a leading cause of end-stage renal disease (ESRD) (1). Characterized by a progressive increase in proteinuria and a persistent decline in renal function, DN culminates in irreversible renal damage, severely compromising patients’ quality of life. With the global diabetic population projected to rise from 537 million to 783 million within the next 24 years, the prevalence of DN is expected to escalate correspondingly (2). At the heart of DN’s pathophysiology is a microinflammatory state, underscored by an innate immune response that perpetuates inflammation in both circulating blood and renal tissues (3). Central to this response are inflammasomes—cytoplasmic multi-protein complexes that activate inflammatory caspase-1, thus playing a pivotal role in the innate immune system (4). Among these, the NOD-like receptor pyrin domain-containing 3 (NLRP3) inflammasome stands out as a key player, extensively implicated in the orchestration of inflammatory responses through the formation and release of cytokines such as interleukin-1β (IL-1β) and IL-18 (5). Consequently, NLRP3’s involvement extends to a myriad of chronic inflammatory conditions, including DN. Current research underscores the close association between NLRP3 inflammasome activity and DN progression, highlighting its significance in understanding and addressing this debilitating complication of DM.

## 2 Overview of NLRP3 inflammasome

The NLRP3 inflammasome is a cytoplasmic multi-protein complex pivotal in innate immunity, comprising the NLRP3 protein, the apoptosis-associated speck-like protein containing a CARD (ASC), and the cysteine aspartic acid protease 1 precursor (pro-caspase-1). NLRP3 serves as a crucial regulatory protein within this complex, featuring a C-terminal leucine-rich repeat (LRR) domain, a central NACHT domain that triggers proinflammatory signaling, and an N-terminal pyrin (PYD) domain for recruiting proteins to assemble the inflammasome (6). ASC acts as an adaptor molecule with its N-terminal PYD and C-terminal caspase activation and recruitment domain (CARD), bridging the NLRP3 protein with caspase-1 (7). Upon sensing specific stimuli, NLRP3 acts as a scaffold to bind ASC, which subsequently recruits and processes pro-caspase-1 into its active form, caspase-1. Caspase-1 then catalyzes its own cleavage into a heterodimer composed of P10 and P20 subunits (8). This activation sequence facilitates the maturation and secretion of inflammatory cytokines, notably IL-1β and IL-18, from their precursors (pro-IL-1β and pro-IL-18), culminating in an inflammatory response.

The activation of the NLRP3 inflammasome extends beyond immune cells such as macrophages and dendritic cells to include intrinsic renal cells, underscoring its broad involvement in renal disease pathogenesis (9). Various stimuli can trigger NLRP3 inflammasome activation, including ion fluxes (10), reactive oxygen species (ROS) (11), mitochondrial dysfunction (12), and lysosomal damage (13).

One notable signaling agent for NLRP3 inflammasome activation is perforin, which compromises cell membrane integrity, leading to intracellular potassium (K^+^) efflux and enabling extracellular molecules like lipopolysaccharide (LPS) to enter the cell (14). This process highlights the critical role of K^+^ in the regulation of the NLRP3 inflammasome. Similarly, respiratory chain inhibitors can induce mitochondrial dysfunction and elevate ROS production, thereby promoting NLRP3 activation. Conversely, the utilization of ROS scavengers or NADPH oxidase inhibitors markedly inhibits this activation process (15).The release of lysosomal histone B emerges as a crucial step for IL-1β production, though it is not necessary for the generation of IL-1β precursors. Histone B facilitates inflammasome activation via ROS mechanisms (16). Moreover, lysosomal damage, potentially leading to the release of lysosomal calcium (Ca^2+^), is implicated in the activation of the NLRP3 inflammasome, further illustrating the complex interplay of cellular events leading to inflammatory responses in renal disease (17).

The classical pathway of NLRP3 inflammasome activation is dependent on caspase-1, encompassing two phases: initiation and activation (18). Phase 1 involves Damage-Associated Molecular Patterns (DAMPs), which activate the nuclear transcription factor-κB (NF-κB) through the pattern recognition receptor family of Toll-like receptors (TLRs), tumor necrosis factor-α (TNF-α) receptor, etc. This activation induces the synthesis and accumulation of NLRP3, pre-interleukin-18 (IL-18) mRNA, pro-interleukin-1α (pro-IL-1α), and pre-interleukin-1β (IL-1β) mRNA (19). Phase 2 is triggered by activation signals from ATP, bacteria, and uric acid crystals, leading to the activation of NLRP3 inflammasomes. This activation promotes the transcriptional expression of pro-IL-1β and pro-IL-18, culminating in the formation and secretion of the inflammatory factors IL-1β and IL-18 (20). Consequently, NF-κB is considered a critical mediator in the activation of NLRP3 inflammasomes due to its major role in pro-inflammatory cytokine mediation (21).

## 3 Role of NLRP3 inflammatory vesicles in diabetic nephropathy

**Figure 1:**
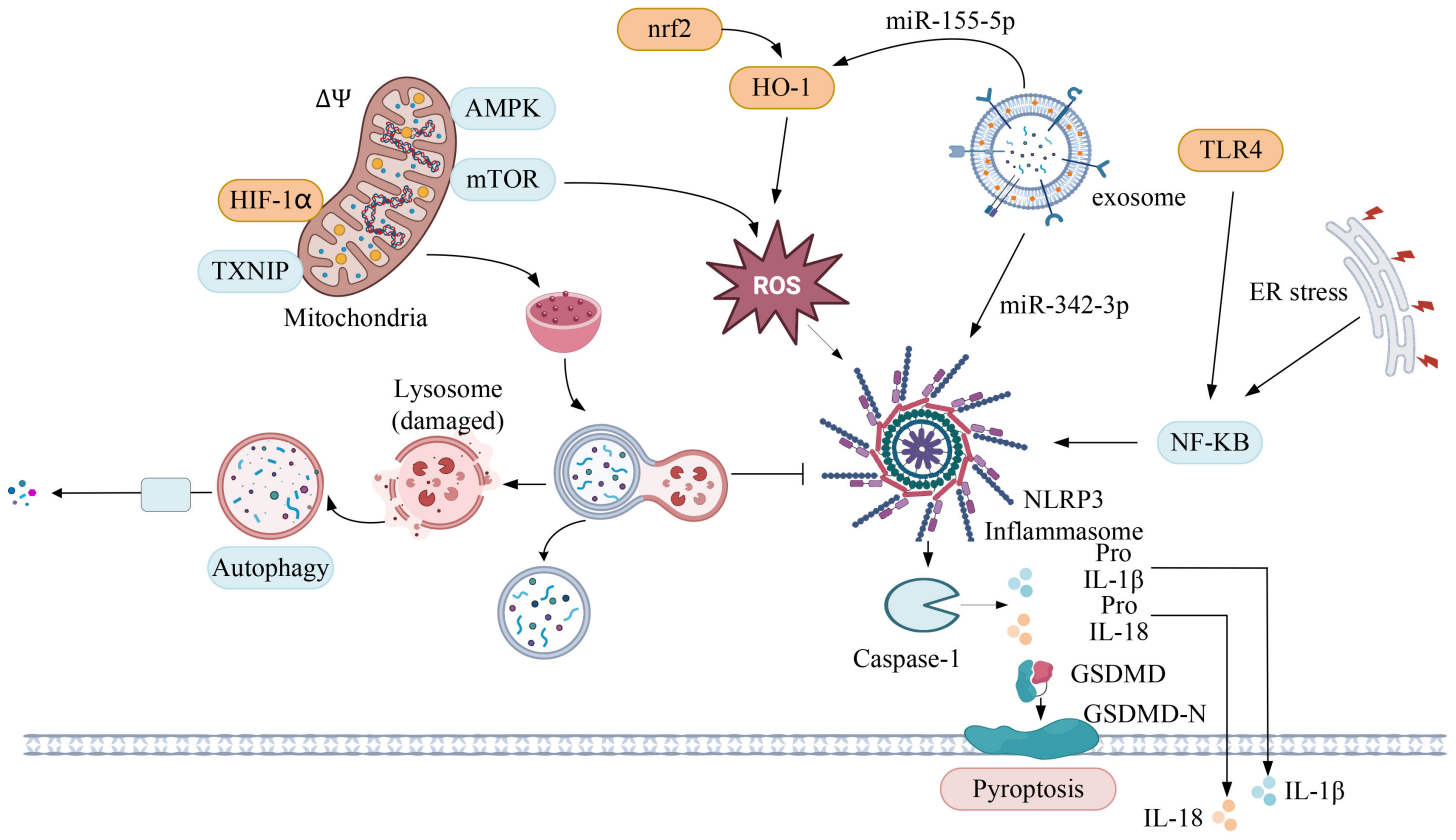
The role of NLRP3 inflammatory vesicles in diabetic nephropathy.

Diabetic nephropathy (DN) is a severe complication of diabetes mellitus (DM), characterized by progressive proteinuria, renal impairment, and varying degrees of renal tissue fibrosis or glomerulosclerosis, potentially leading to end-stage renal disease. Increasing evidence underscores the critical role of inflammatory cell infiltration in DN pathogenesis. Activation of inflammatory mediators, such as C-reactive protein (CRP), monocyte chemotactic protein-1 (MCP-1), and inflammatory vesicles, drives macrophage infiltration, tubular fibrosis, and accelerates glomerulosclerosis (22). Recent studies have spotlighted the NLRP3 inflammasome’s pivotal role in renal inflammation, contributing to renal pathological lesions and injury. See **Figure 1**.

### 3.1 Mediation of cellular pyroptosis

Introduced in 2001, “cellular pyroptosis” denotes a pro-inflammatory programmed cell death (23). In a sustained high-glucose environment, NLRP3 collaborates with ASC and proCaspase-1 to form an inflammatory complex, subsequently activating Caspase-1. Activated Caspase-1 enhances the maturation of pro-IL-1/pro-IL-18, releasing abundant inflammatory cytokines, including IL-1β and IL-18 (24). Simultaneously, Caspase-1 cleaves Gasdermin D (GSDMD). The N-terminus of GSDMD, possessing pore-forming activity, attaches to the cell membrane, creating large pores (25). This leads to cell membrane rupture, discharge of cellular contents, and release of inflammatory factors, culminating in cellular pyroptosis. Research highlights the therapeutic potential of modulating this pathway in DN. Zhang et al. (26) demonstrated that MCC950, a potent NLRP3 inhibitor, could mitigate podocyte injury and renal fibrosis by targeting the NLRP3/caspase-1/IL-1β axis. Similarly, Wang et al. (27) observed that inhibiting TLR4/NF-κB signaling could reverse the upregulation of GSDMD-NT under hyperglycemic conditions, reducing IL-1β release, alleviating proteinuria, and decelerating renal injury. This suggests that TLR4/NF-κB signaling plays a crucial role in GSDMD-mediated damage in DN renal tubular cells. Further studies have implicated the hypoxia-inducible factor 1α (HIF-1α) and glycogen synthase kinase 3β (GSK-3β) signaling pathway in the regulation of inflammatory responses and cell death in DN (28). Specifically, high glucose conditions that elevate HIF-1α expression in renal proximal tubular epithelial cells can be counteracted by GSK-3β inhibition, leading to reduced NLRP3 and caspase-1 activation, and subsequently, lower levels of IL-1β and IL-18.

Moreover, emerging evidence suggests that endoplasmic reticulum stress may contribute to DN pathogenesis through the NF-κB/NLRP3 pathway, offering additional targets for therapeutic intervention (29). Collectively, these findings underscore the complexity of DN at the molecular level and the potential for targeting the NLRP3 inflammasome and associated signaling pathways to ameliorate DN progression.

### 3.2 Involvement in oxidative stress

Oxidative stress plays a pivotal role in the development of DN. The *in vivo* overproduction of reactive oxygen species (ROS) is a critical driver in the activation of NLRP3 inflammatory vesicles. The nuclear transcription factor erythroid 2-related factor 2 (Nrf2), pivotal in regulating oxidative stress, dissociates from Kelch-like ECH-associated protein 1 (Keap1) and translocates to the nucleus (30). Here, it activates downstream target genes, such as heme oxygenase-1 (HO-1) and superoxide dismutase (SOD), effectively mitigating oxidative stress. Recent studies underscore the ability of the Nrf2/HO-1 signaling pathway to inhibit NLRP3 inflammatory vesicle activation, thereby attenuating podocyte injury and improving DN-related renal function and histopathology (31).

Mitochondrial ROS further stimulate the localization of NLRP3 to mitochondria-associated endoplasmic reticulum membranes (MAMs), facilitating the recruitment of ASC and the assembly with NLRP3 and pro-Caspase-1 to form the NLRP3 inflammasome. This assembly promotes the release of inflammatory cytokines, such as IL-1β and IL-18, which in turn trigger the production of pro-fibrotic factors like TGF-β, fostering renal fibrosis (32). Han et al. (33) highlighted that an overproduction of mitochondrial ROS leads to a reduction in thioredoxin (TRX) levels and an increase in thioredoxin-interacting protein (TXNIP), alongside elevated expression of NLRP3, IL-1β, and TGF-β. The application of the mitochondrial ROS antioxidant MitoQ has been shown to inhibit the dissociation of TRX from TXNIP, blocking the TXNIP-NLRP3 interaction and reducing the release of IL-1β. This indicates that the mitochondrial ROS-TXNIP/NLRP3/IL-1β axis may be responsible for oxidative damage in renal tubules. Moreover, in mice, high glucose levels can activate NLRP3 inflammatory vesicles in glomerular podocytes by stimulating nicotinamide adenine dinucleotide phosphate (NADPH) oxidase, leading to an inflammatory response and podocyte injury (34). Thus, the suppression of intracellular ROS levels can serve as a strategy to prevent NLRP3 inflammasome activation.

### 3.3 Regulation of podocyte autophagy

Autophagy, a highly conserved lysosomal degradation mechanism, plays a critical role in cellular defense. It removes damaged organelles and dysfunctional proteins, thereby safeguarding cells from injury. This process is essential for maintaining the normal function of glomerular podocytes, key components in kidney filtration (35). A majority of studies have highlighted a deficiency in podocyte autophagy in the context of diabetic nephropathy (DN). Overactivation of the mechanistic target of rapamycin (mTOR) pathway, primarily via its upstream phosphatidylinositol 3-kinase/protein kinase B (PI3K/Akt) signaling, has been identified as a central factor leading to impaired autophagy in podocytes (36). This impairment contributes to excessive matrix accumulation and accelerates renal fibrosis.AMP-activated protein kinase (AMPK) serves as a crucial nutrient-sensitive pathway regulating autophagy in DN-afflicted podocytes. Activated AMPK can induce autophagy by inhibiting mTOR complex 1 (mTORC1) through the phosphorylation of the TSC1-TSC2 complex, thus offering a protective effect against kidney damage (37). Recent investigations have elucidated the interaction between autophagy and the NLRP3 inflammasome in DN. On one side, studies, such as those conducted by Wang et al., demonstrate that modulating the AMPK/mTORC1/NLRP3 signaling axis in diabetic kidneys can suppress NLRP3 inflammasome-mediated cellular death and mitigate renal fibrosis (38). Conversely, research by Hao et al. (39) reveals that NLRP3 inflammatory vesicles can adversely affect podocyte autophagy, leading to a deficiency in this protective mechanism. However, under high glucose conditions, inhibiting NLRP3 activation can stimulate podocyte autophagy. Therefore, targeting autophagy or inflammatory pathways offers a promising approach to delay renal injury in diabetic nephropathy, showcasing the intricate balance between cellular degradation processes and inflammatory responses in disease progression.

### 3.4 NLRP3 and exosomes

Exosomes, small lipid bilayer vesicles with diameters ranging from 40 to 160 nm, are carriers of diverse biomolecules, including proteins, DNA fragments, messenger RNA (mRNA), microRNA (miRNA), circular RNA (circRNA), and transcription factors (40). These vesicles play a pivotal role in intercellular communication. Liu et al. (41) observed that exosomes derived from macrophages stimulated by high glucose levels significantly increased 24-hour urinary protein, serum blood urea nitrogen (BUN), and serum creatinine (Scr) levels in DN mice. Additionally, these exosomes upregulated the expression of inflammatory markers such as NLRP3, ASC, Caspase-1, and IL-1β. This suggests that macrophage-derived exosomes under high glucose conditions exacerbate the inflammatory response and activate the NLRP3 inflammasome, leading to abnormal proliferation of mesangial cells, excessive extracellular matrix accumulation, and accelerated renal fibrosis. Furthermore, Zheng et al. (42) discovered through RNA sequencing that miR-342–3p was significantly down-regulated in the kidneys of DN rats. They also found that exosomes from human umbilical cord mesenchymal stem cells (UC-MSCs) could deliver miR-342–3p, resulting in reduced expression of ASC and IL-1β in the kidney. This indicates that UC-MSCs may protect against renal tubular epithelial cell death in DN by releasing miR-342–3p, which targets the NLRP3/Caspase-1 signaling pathway. Hence, future therapeutic strategies aimed at modulating exosomal contents could offer a novel approach to mitigating renal pathological changes by inhibiting NLRP3 inflammasome activation in diabetic nephropathy.

## 4 Intervention of Chinese herbal medicine on NLRP3 inflammatory vesicles in diabetic nephropathy

### 4.1 Traditional Chinese medicine monomers

#### 4.1.1 Astragaloside IV

Astragaloside IV, a lanolinol-form tetracyclic triterpenoid saponin, is a principal active component of Astragalus, widely recognized in traditional Chinese medicine. Pharmacological research underscores its multifaceted benefits, including anti-inflammatory, antioxidant, hypoglycemic, hypolipidemic, and antifibrotic effects (43). AS-IV’s efficacy in DN encompasses the amelioration of oxidative stress, inflammation reduction, and endoplasmic reticulum stress mitigation (44). In db/db mice, AS-IV administration notably decreased urinary protein, serum creatinine (Scr), and blood urea nitrogen (BUN) levels, alongside reducing renal tissue expressions of NLRP3, caspase-1, and IL-1β. Additionally, AS-IV thwarted the high glucose-induced activation of NLRP3 in mouse podocytes *in vitro*, suggesting a protective role against renal function deterioration and podocyte injury via dampening NLRP3 inflammasome-mediated inflammation (45). Recent studies (46) have demonstrated that Astragaloside IV (AS-IV) can suppress the expression of NLRP3, Caspase-1, GSDMD, IL-1β, and IL-18, thereby mitigating podocyte cell death in diabetic nephropathy (DN). This effect is mediated through the upregulation of deacetylase 6 (SIRT6) and the downregulation of the hypoxia-inducible factor 1 α subunit (HIF-1α), indicating a potential therapeutic pathway for addressing the underlying mechanisms of DN. Moreover, AS-IV engages the NF-κB/NLRP3 signaling pathway to elevate endogenous klotho protein expression, thus offering a protective shield for DN-affected glomerular podocytes in hyperglycemic conditions (47).

#### 4.1.2 Triptolide

Triptolide (TP), a natural diterpene triepoxide, is a prominent active component derived from the traditional Chinese herb Lei Gong Teng (Thunder God Vine). Known for its potent anti-inflammatory properties, TP has been extensively documented to modulate the inflammatory response by inhibiting the secretion of inflammatory cytokines and inducing apoptosis in fibroblasts (48). Research underscores TP’s protective effects against DN, particularly in safeguarding podocytes from damage and reducing proteinuria levels (49). Wu et al. (50) have demonstrated that TP mitigates high glucose-induced epithelial-mesenchymal transition (EMT) in podocytes, a critical process in DN pathogenesis, by attenuating the activation of NLRP3 inflammatory vesicles. This action of TP is pivotal in preventing podocyte pyroptosis—a form of programmed cell death associated with inflammation—and improving the structural and functional integrity of the kidneys in DN. One mechanism through which TP exerts these protective effects involves the upregulation of nuclear factor-erythroid 2-related factor 2 (Nrf2) and heme oxygenase-1 (HO-1) protein expression, alongside a reduction in reactive oxygen species (ROS) levels (51). Through these pathways, TP not only inhibits NLRP3 inflammasome activation but also ameliorates oxidative stress, underscoring its therapeutic potential in DN management.

#### 4.1.3 Ginsenosides

Ginsenosides, the active monomeric ingredients of ginseng, exhibit a broad spectrum of pharmacological properties, including antioxidant, anti-inflammatory, hypoglycemic, and anti-aging effects (52). Among the various ginsenosides, Compound K stands out for its significant improvement in kidney function in DN by reducing the expression of inflammatory cytokines such as IL-1β and IL-18. This effect is largely attributed to its ability to inhibit the TXNIP-mediated activation of NLRP3 inflammatory vesicles (53). Ginsenoside Rg1 has been identified as another potent compound, offering protection against hyperlipidemia-induced injury in DN podocytes through the mTOR/NF-κB/NLRP3 signaling axis (54). This pathway plays a crucial role in inhibiting cell death and ameliorating renal damage. Similarly, Ginsenoside Rg5 has been shown to diminish inflammatory responses by inhibiting the activation of the NLRP3 inflammasome, consequently reducing blood glucose, creatinine, and urea nitrogen levels in mice with diabetic nephropathy (DN) (55). Furthermore, it significantly ameliorates renal pathology in these diabetic mice. Moreover, Ginsenoside Rg3 has demonstrated the capacity to block IL-1β secretion and caspase-1 activation by inhibiting NLRP3 inflammasome activation in both human and mouse macrophages (56). This mechanism further underscores the therapeutic potential of ginsenosides in mitigating inflammatory processes implicated in the progression of DN.

#### 4.1.4 Geniposide

Geniposide, a cyclen terpene glycoside, stands as a principal active component of the Gardenia plant, revered in Chinese medicine for its broad pharmacological spectrum. Experimental evidence supports GE’s anti-inflammatory, antioxidant capabilities, alongside its roles in protecting the central nervous system and modulating immunity (57). Geniposide (GE) has been demonstrated to attenuate monocyte and T-lymphocyte infiltration, suppress the secretion of pro-inflammatory cytokines, including tumor necrosis factor-α (TNF-α), interleukin-1 (IL-1), and IL-6, and inhibit the NF-κB signaling pathway (58). These actions collectively contribute to the enhancement of both structure and function in the kidneys of rats afflicted with diabetic nephropathy. A critical mechanism through which GE exerts its therapeutic effect involves the inhibition of NLRP3-mediated cellular processes. GE significantly diminishes the protein expression along the AMPK/SIRT1/NF-κB pathway, thereby blocking NLRP3-mediated cellular pyroptosis and oxidative stress, crucial contributors to the pathogenesis of DN (59). Moreover, crocetin, another active ingredient derived from Gardenia jasminoides, complements GE’s anti-inflammatory profile. Crocetin notably suppresses the production of reactive oxygen species (ROS) and pro-inflammatory factors, including TNF-α, IL-1β, and IL-18, by inhibiting NLRP3 inflammatory vesicle expression in renal tissues of DN rats (60). This collective action significantly ameliorates renal fibrosis, highlighting the potential of GE and related compounds in the management of DN.

#### 4.1.5 Berberine

Berberine, the principal bioactive component of the traditional Chinese herb Bergenia lutea, known for its efficacy in treating inflammatory diseases and diabetes mellitus, has garnered attention for its renal protective properties (61). Research has illuminated BBR’s capacity to ameliorate renal impairment and counteract podocyte dysfunction via key signaling pathways, including phosphatidylinositol 3-kinase/protein kinase B (PI3K-Akt) (62), Toll-like receptor 4/nuclear factor kappa-light-chain-enhancer of activated B cells (TLR4/NF-κB) (63), and transforming growth factor-β/Smad3 (64). These mechanisms collectively underscore BBR’s protective role in diabetic nephropathy. The epithelial-to-mesenchymal transition (EMT) process, a pivotal event in the initiation of cellular matrix accumulation and renal fibrosis (65), has been identified as a critical target for DN management (66). BBR’s ability to mitigate high glucose-induced EMT and renal interstitial fibrosis highlights its potential as a therapeutic agent, chiefly through the inhibition of the NLRP3 inflammasome’s activation. Furthermore, studies by Ma et al. (67) and Ding et al. (68) have demonstrated BBR’s effectiveness in reducing blood glucose levels, serum creatinine, and urea nitrogen, thereby improving the renal pathology in DN models. The underlying mechanism attributed to BBR’s efficacy involves the suppression of NLRP3-Caspase-1-GSDMD signaling-mediated cell death, augmented by the upregulation of the antioxidant transcription factor nuclear factor erythroid 2-related factor 2 (Nrf2). This modulation results in decreased release of inflammatory cytokines and alleviation of oxidative stress, offering a promising avenue for DN treatment.

#### 4.1.6 Other herbal monomers

Recent research has highlighted the efficacy of several herbal monomers in delaying kidney injury progression through their antioxidant properties. High glucose conditions induce excessive production of reactive oxygen species (ROS), leading to oxidative stress. Under such stress, the nuclear factor erythroid 2-related factor 2 (Nrf2) dissociates from its inhibitor, Kelch-like ECH-associated protein 1 (KEAP1), and translocates to the nucleus (69). There, Nrf2 activates the transcription of downstream antioxidant enzymes like heme oxygenase-1 (HO-1), effectively mitigating oxidative stress. The Nrf2 pathway is noted for its dual antioxidant and anti-inflammatory functions, offering a protective mechanism against DN progression. Syringaresinol, for example, can alleviate DN by suppressing NLRP3 expression through the Nrf2-mediated antioxidant pathway (70). The interaction between thioredoxin-interacting protein (TXNIP) and ROS is crucial for the activation of NLRP3 inflammatory vesicles, representing a key target for therapeutic intervention (71). Compounds such as Tanshinone IIA (72) and Salidroside (73) have been shown to inhibit cellular death and delay DN progression by targeting the TXNIP/NLRP3 pathway.Calycosins, principal components of Astragalus, exhibit multiple pharmacological effects, including the ability to improve renal function in DN. These effects are achieved by modulating the NF-κB/p65/NLRP3/TXNIP signaling pathway, reducing lactate dehydrogenase activity, and attenuating cellular damage (74). Andrographolide further demonstrates the potential to inhibit NLRP3 inflammasome activation and prevent renal tubular epithelial cell apoptosis by ameliorating mitochondrial dysfunction (75). Breviscapine has been found to reduce inflammasome activation by targeting high glucose-induced NF-κB signaling, decreasing cellular pyroptosis, and modulating the expression of α-smooth muscle actin (α-SMA) and podoplanin (76). Curcumin, known for its broad-spectrum therapeutic effects, inhibits the upregulation of type IV collagen and fibronectin in the renal cortex, reduces membrane matrix expansion, and decreases albuminuria levels in db/db mice, likely through the inhibition of NLRP3 inflammatory vesicle activity (77) (**Table 1**).

**Table 1:** The molecular mechanism of Chinese medicine monomer regulating NLRP3 inflammasome in DN.

Chinese Medicine Monomer	Model	Mechanism	Reference
**Astragaloside IV**	db/db mice; Podocytes	Inhibits NLRP3 inflammasome-mediated inflammation	(45)
	Male SD rats; Podocytes	Improves podocyte pyroptosis in DKD by regulating the SIRT6/HIF-1α pathway	(46)
	SD rats; Mouse podocytes	Increases Klotho expression via the NF-κB/NLRP3 axis	(47)
**Triptolide**	Podocytes of mice	Inhibits NLRP3 inflammasome activation and ameliorates podocyte EMT	(50)
	DN mouse models; MPC5 podocytes	Alleviates podocyte injury in DN by inhibiting oxidative stress and pyroptosis via the Nrf2/ROS/NLRP3 axis	(51)
**Ginsenoside Metabolite Compound K**	Mice; Rat glomerular mesangial cell line HBZY-1	Inhibits ROS-mediated activation of the NLRP3 inflammasome and NF-κB/p38 signaling pathway	(53)
**Ginsenoside Rg1**	Male SD rats; Podocyte cell line BNCC337685	Inhibits pyroptosis through the mTOR/NF-κB/NLRP3 axis	(54)
**Ginsenoside Rg5**	C57BL/6 diabetic mice	Inhibits oxidative stress and NLRP3 inflammasome activation to reduce inflammatory responses	(55)
**Geniposide**	C57/B6 mice; HG-induced podocyte model	Reduces the expression of AMPK/SIRT1/NF-κB pathway proteins and inhibits the activation of the NLRP3 inflammasome	(59)
**Crocin**	Sprague–Dawley male rats	Inhibits the activation of the NLRP3 inflammasome and reduces ROS, TNF-α, IL-1β, and IL-18	(60)
**Berberine**	SD rats; HK-2 cells	Inhibits high glucose-induced EMT and renal interstitial fibrosis by suppressing the NLRP3 inflammasome	(67)
	Male golden hamsters	Upregulates the expression of antioxidant factor Nrf2 and inhibits NLRP3-Caspase-1-GSDMD signal-mediated pyroptosis	(68)
**Syringaresinol**	C57BL/6J mice	Inhibits the NLRP3/Caspase-1/GSDMD pyroptosis pathway by upregulating NRF2 signaling in DN	(70)
**Tanshinone IIA**	db/db mice; Human renal glomerular endothelial cells (HRGECs)	Delays the progression of DKD by inhibiting pyroptosis via the Txnip/NLRP3 inflammasome	(72)
**Salidroside**	Rat glomerular mesangial cell line HBZY-1	Alleviates HG-induced oxidative stress and ECM accumulation in rat glomerular mesangial cells by the TXNIP-NLRP3 inflammasome pathway	(73)
**Calycosin**	Male Sprague-Dawley rats	Hinders the development of DN through modulation of the NF-κB/p65/NLRP3/TXNIP inflammasome signaling pathway	(74)
**Andrographolide**	Human tubular epithelial cells (HK-2 cells)	Male C57BL/6 mice Suppresses mitochondrial dysfunction and NLRP3 inflammasome activation	(75)
**Breviscapine**	Male C57BL/6J wild-type mice; MPC5 podocytes	Alleviates podocyte injury by inhibiting NF-κB/NLRP3-mediated pyroptosis in diabetic nephropathy	(76)
**Curcumin**	Male C57BL/KsJ db/db mice; HK-2 cells	Suppresses NLRP3 inflammasome signaling	(77)

### 4.2 Chinese medicine compound prescription

The practice of Chinese medicine compounding, characterized by its utilization of multiple components, pathways, and targets, has demonstrated significant therapeutic efficacy in managing diabetes mellitus (78) and its complications (79). Among these compound prescriptions, the Yi Shen Pai Du Formula (YSPDF) stands out for its composition and potential benefits. YSPDF comprises a blend of Astragali radix (Huang Qi), Rhei radix et rhizome (Da Huang), Hirudo (Shui Zhi), and Bombyx batrytocatus (Jiang Can), each selected for their unique pharmacological properties.Studies indicate that YSPDF (80) can notably reduce oxidative stress and inflammatory factor release, thereby alleviating renal fibrosis in db/db mice models. Further *in vitro* analyses suggest that YSPDF may prevent the epithelial-mesenchymal transition (EMT) and inhibit NLRP3 inflammatory vesicle formation in renal tubular epithelial cells under high glucose conditions, primarily through the activation of the Nrf2 pathway. This indicates a multifaceted approach to modulating cellular processes pivotal in the progression of diabetic nephropathy. Hirudin, derived from the medicinal leech’s salivary glands, contributes significantly to YSPDF’s efficacy. With its anticoagulant, antifibrotic, antithrombotic, and anti-inflammatory properties, hirudin offers substantial kidney protection (81). Specifically, it suppresses the expression of protease-activated receptor 1 via the S1P/S1PR2/S1PR3 signaling pathway, curbing TGF-β-mediated fibrosis in renal proximal tubular epithelial cells (82). This mechanism underscores the complex yet highly targeted action of YSPDF and similar Chinese medicine compounds in combating renal pathologies associated with diabetes.

Alterations in the gut microbiota and its metabolites, including short-chain fatty acids (SCFAs), trimethylamine oxide (TMAO) (83), and secondary bile acids (84), play a pivotal role in the exacerbation of renal disease progression and impact distant organs. Notably, excessive intestinal TMAO has been linked to renal tubulointerstitial fibrosis in diabetes mellitus (DM) patients, enhancing the expression of pro-fibrotic genes and markers of kidney injury (85). Moreover, TMAO is known to elevate intracellular mitochondrial reactive oxygen species (mROS) levels, thereby activating NLRP3-mediated cellular death (86).In response to these challenges, the Zuogui-Jiangtang-Yishen (ZGJTYS) decoction, an optimization of the classic Chinese herbal formula Zuoguiwan, targets the mROS-NLRP3 axis (87). ZGJTYS regulates this axis to inhibit the activation of cellular focal death induced by intestinal-derived TMAO, thereby ameliorating glucose-lipid metabolism disorders and improving renal function (88). This approach not only delays renal pathological changes but also prevents DN progression.

Additionally, the Yiqi-Huoxue-Jiangzhuo formula (YHJF), validated by Guang’anmen Hospital of the China Academy of Traditional Chinese Medicine (89), has shown efficacy in treating CKD and its complications (90). YHJF’s cardioprotective effects in mice with CKD are attributed to gut microbiota modulation and NLRP3 inflammatory vesicle inhibition (91). The Tongluo Yishen (TLYS) decoction, comprising Salvia miltiorrhiza and Fangji, further illustrates the therapeutic potential of Chinese medicine compounds. TLYS has demonstrated the ability to ameliorate renal fibrosis by modulating NLRP3-mediated cell death, showcasing significant results after just two weeks of intervention in rat models (92) (**Table 2**).

**Table 2:** The molecular mechanism of Chinese herbal compound regulating NLRP3 inflammasome in DN.

Chinese Herbal Compound	Model	Dosage	Mechanism	Reference
**Yi Shen Pai Du Formula (YSPDF)**	db/db mice	0.4 ml YSPDF once daily;	Inhibits oxidative stress, inflammation, and EMT, potentially through activation of the Nrf2 pathway	(80)
	HK-2 cells	0.4mg/mL YSPDF		
**Zuogui-Jiangtang-Yishen Decoction**	GK rats	ZG-H:19.9 g·kg-1·d ZGJTYS ZG-M:9.9 g·kg-1·d ZG-L:4.9 g·kg-1·d	Inhibits activation of pyroptosis by gut-derived TMAO via the mROS-NLRP3 axis to prevent DKD	(88)
	HK-2 cells	15% ZGJTYS-drug serum		
**Yiqi Huoxue Jiangzhuo Formula (YHJF)**	C57BL/6J mice	High-dose 23.6g/kg/dYHJF; medium-dose 11.8 g/kg/d; low-dose5.9 g/kg/d	Inhibits NLRP3 inflammasome activation	(91)
**Tongluo Yishen Decoction (TLYS)**	UUO rat	0.8 g/100 (g·d) TLYS	Exerts renoprotective effects by inhibiting NLRP3-mediated pyroptosis	(92)
	NRK-52E cells	high, middle and low doses of TLYS were 500μg/ml, 200μg/ml, and 100μg/ml		

### 4.3 Proprietary Chinese medicines

#### 4.3.1 Huangkui capsule

The Huangkui capsule, a modern proprietary Chinese medicine extracted from Abelmoschus manihot (L.), has garnered approval from the State Food and Drug Administration of China (State Pharmaceutical License Z19990040) (93). Its widespread use in managing diabetic nephropathy (DN) underscores its therapeutic significance. Through high-performance liquid chromatography (HPLC) analysis, the capsule has been identified to contain seven classes of flavonoids, including rutin, chrysin, isoquercitrin, populin, and quercetin (94). These flavonoids are recognized for their potent antioxidant and antilipidemic properties (95).

In the context of hyperglycemic conditions, rutin demonstrates the capability to diminish reactive oxygen species (ROS) by suppressing the Nox4 gene expression, leading to the downregulation of TXNIP, NLRP3, and caspase-1 expression (96). Furthermore, comprehensive extracts of the Huangkui capsule have been shown to mitigate renal tubular injury. This protective effect is attributed to the inhibition of NLRP3 inflammatory vesicle activation via the ERK1/2 signaling pathway (97).

Mechanistic studies increasingly affirm the capsule’s efficacy in improving proteinuria, evidenced by the downregulation of biomarkers such as col4a3, slc5a2, slc34a1, slc12a3, and slc4a1 in the glomeruli and proximal and distal tubules of db/db mice kidneys (98). Additionally, the Huangkui capsule counteracts tubular epithelial-to-mesenchymal transition (EMT) induced by NLRP3 inflammatory vesicle activation through the TLR4/NF-κB signaling pathway, thus contributing to the alleviation of renal fibrosis (99).

#### 4.3.2 Other proprietary Chinese medicines

In addition to the Huangkui capsule, other proprietary Chinese herbal formulations have shown promise in mitigating renal injury through various mechanisms:

Suyin Detoxification Granule (100): This herbal granule plays a pivotal role in attenuating renal injury by inhibiting cellular pyroptosis and the epithelial-mesenchymal transition (EMT). Its mechanism involves the down-regulation of the MAVS/NLRP3 signaling pathway, highlighting a novel approach to modulating inflammatory processes in the kidney.

Yishen Capsule (101): Known for reducing microalbuminuria and alleviating pathological changes in DN rats, the Yishen Capsule targets the NOD-like receptor signaling pathway. By modulating this pathway, the capsule effectively addresses underlying mechanisms contributing to renal damage.

San-Huang-Yi-Shen Capsules (102): These capsules have been reported to promote PINK1/Parkin-mediated mitochondrial autophagy while inhibiting NLRP3 inflammatory vesicle activation. Such actions not only ameliorate mitochondrial injury but also curb the inflammatory response, offering a dual therapeutic benefit. (**Table 3**)

**Table 3:** The molecular mechanism of proprietary Chinese Medicine regulating NLRP3 inflammasome in DN.

Proprietary Chinese Medicine	Model	Dosage	Mechanism	Reference
**Huangkui Capsule**	male SD rats	2g/kg/day HKC	Inhibits NLRP3 vesicle via TLR4/NF-κB pathways	(99)
**Suyin Detoxification Granule**	male SD rats	high-dose SDG group(10 g/kg/d SDG), low-dose group(5 g/kg/d SDG)	Inhibits cellular pyroptosis and EMT by downregulating the MAVS/NLRP3 signaling pathway	(100)
	HK-2 cells	low-dose: 5 g/L SDP; medium-dose: 10 g/L; high-dose: 20 g/L		
**Yishen Capsule**	male SD rats	1.25 g/kg/d Yishen capsule	Targets the NOD-like receptor signaling pathway	(101)
**San-Huang-Yi-Shen Capsules**	male SD rats	low-dose: 0.81g/kg SHYS high-dose: 1.62g/kg SHYS	Activates PINK1/Parkin-mediated mitochondrial autophagy; Inhibits NLRP3 inflammatory vesicle activation	(102)

**Figure 2:**
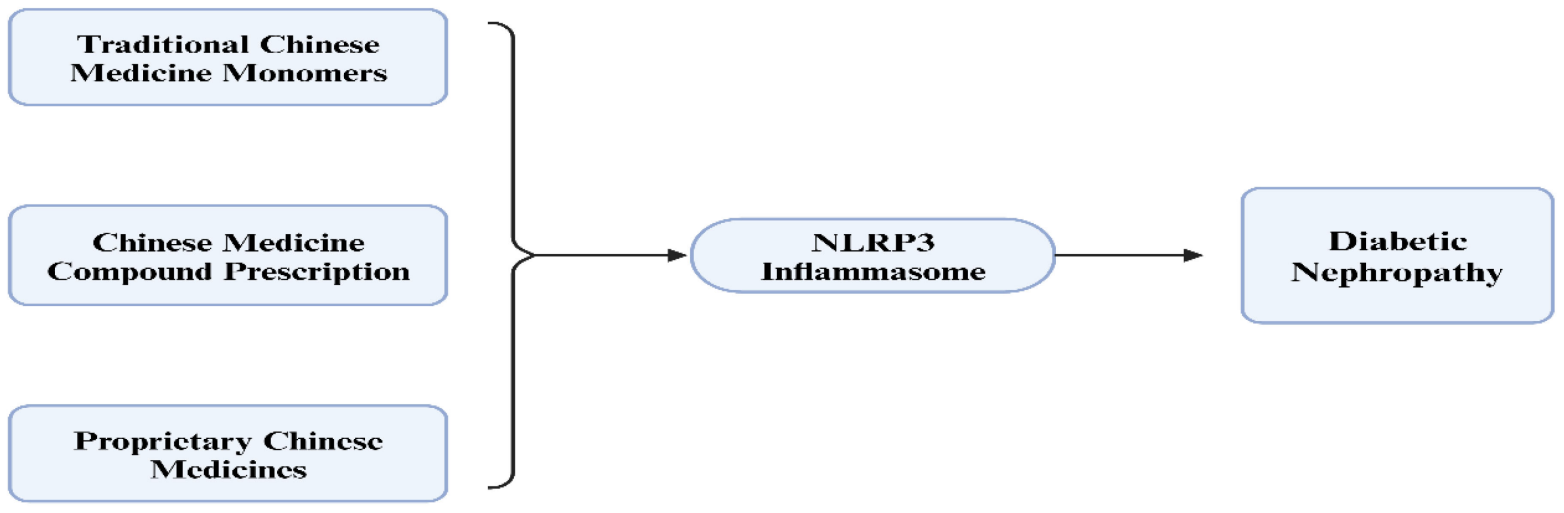
Intervention of Chinese herbal medicine on NLRP3 inflammatory vesicles in diabetic nephropathy.

Collectively, these studies underscore the ability of traditional Chinese medicine to regulate NLRP3 vesicle activation and improve diabetic nephropathy outcomes through multiple pathways (**Figure 2**). The diverse mechanisms of action exhibited by these proprietary formulations reflect the comprehensive approach of traditional Chinese medicine in targeting the multifaceted nature of DN.

## 5 Summary and outlook

The NLRP3 inflammasome is a crucial component of the body’s innate immune system, with its activation mechanisms widely attributed to ion flux, mitochondrial reactive oxygen species (ROS), and lysosomal damage. Extensive research has established that the activation n of the NLRP3 inflammasome contributes significantly to renal inflammation, leading to renal impairment. This process is intimately linked with the progression of diabetic nephropathy (DN), influencing the disease through various pathways such as inflammatory responses, oxidative stress, autophagy, and exocytosis. Given the NLRP3 inflammasome’s central role in DN, strategies aimed at targeting the expression of NLRP3 or caspase-1 represent promising therapeutic avenues for managing this condition. The application of multi-target and multi-pathway interventions through Chinese herbs and extracts against NLRP3 inflammatory vesicle activation holds considerable promise in decelerating DN progression. However, the realization of their full potential necessitates further experimental and clinical research. Future studies should focus on identifying Chinese herbs capable of inhibiting NLRP3 inflammatory vesicle activation and elucidating their precise mechanisms of action. Such research endeavors will not only shed light on innovative treatment strategies but also aim to enhance the quality of life for patients suffering from DN.

## Author contributions

JJ: Writing – original draft, Writing – review & editing. MZ: Conceptualization, Funding acquisition, Writing – review & editing.
